# Investigating Whether a Combination of Electro-Encephalography and Gene Expression Profiling Can Predict the Risk of Chronic Pain: A Protocol for an Observational Prospective Cohort Study

**DOI:** 10.3390/brainsci14070641

**Published:** 2024-06-26

**Authors:** Ann-Christin Sannes, Usman Ghani, Imran Khan Niazi, Torgeir Moberget, Rune Jonassen, Heidi Haavik, Johannes Gjerstad

**Affiliations:** 1 Faculty of Health Science, Oslo Metropolitan University, 0890 Oslo, Norway; 2 Department for Research and Development in Mental Health, Akershus University Hospital, 1474 Lørenskog, Norway; 3 Centre for Chiropractic Research, New Zealand College of Chiropractic, Auckland 1060, New Zealandimran.niazi@nzchiro.co.nz (I.K.N.);; 4 Faculty of Health & Environmental Sciences, Health & Rehabilitation Research Institute, AUT University, Auckland 1010, New Zealand; 5 Faculty of Medicine, Aalborg University, 9260 Aalborg, Denmark; 6 Faculty of Health Sciences, Kristiania University College, 0107 Oslo, Norway; 7 Centre for Precision Psychiatry, University of Oslo, 0373 Oslo, Norway

**Keywords:** low back pain, RNA sequencing, electro-encephalography (EEG)

## Abstract

Despite most episodes of low back pain (LBP) being short-lasting, some transition into persistent long-lasting problems. Hence, the need for a deeper understanding of the physiological mechanisms of this is pertinent. Therefore, the aims of the present study are (1) to map pain-induced changes in brain activity and blood gene expression associated with persistent LBP, and (2) to explore whether these brain and gene expression signatures show promise as predictive biomarkers for the development of persistent LBP. The participants will be allocated into three different pain groups (no pain, mild short-lasting, or moderate long-term). One in-person visit, where two blood samples will be collected and sent for RNA sequencing, along with resting 64-channel electro-encephalography measurements before, during, and after a cold pressor test, will be conducted. Thereafter, follow-up questionnaires will be distributed at 2 weeks, 3 months, and 6 months. Recruitment will start during the second quarter of 2024, with expected completion by the last quarter of 2024. The results are expected to provide insight into the relationship between central nervous system activity, gene expression profiles, and LBP. If successful, this study has the potential to provide physiological indicators that are sensitive to the transition from mild, short-term LBP to more problematic, long-term LBP.

## 1. Introduction

Globally, one of the most common musculoskeletal problems is low back pain (LBP), with a global prevalence of 619 million cases [1]. LBP is the leading cause of years lived with disability [1,2], and is most often classified as non-specific LBP [3], implying no pathoanatomical cause for the pain [4,5]. While acute LBP often resolves relatively rapidly, with fairly little impact on long-term health status, the development of chronic or persistent LBP is associated with several serious health consequences [6,7]. Of those experiencing acute pain, approximately 30% will transition to persistent pain [8]. 

Previous evidence shows that persistent pain, lasting 3 months or more [9], is associated with neuronal changes in central nervous system (CNS) and autonomic nervous system (ANS) activity [10,11]. One example is the observed shift in activity from sensory areas to emotional/limbic areas seen in patients with persistent pain [12]. The experience of pain, arising from such areas of the brain, can be altered via bottom–up (e.g., stimulus intensity) or top–down (e.g., expectations)-mediated factors [13]. Thus, physical factors, but also emotional or psychosocial factors such as stress [14], can facilitate functional modifications of the nervous system and have an impact on both the intensity and duration of pain. Stress, which can be elicited both psychologically or from a painful stimulus, activates the hypothalamic–pituitary–adrenal (HPA) axis [15] through the amygdala [16], which, in turn, regulates cortisol levels. A disturbance or a dysregulation in cortisol levels has been found to be associated with stress-related pain disorders [14,15], especially in chronic stress. Psychosocial factors such as catastrophizing [17], depression [18], and anxiety [18,19] have been associated with endogenous pain modulation via descending pain inhibitory pathways leading to an increased experience of pain [17,20]. This may be especially true for persistent pain where tissue damage may no longer be present in the painful area [21]. 

Such functional modifications of the nervous system are called neuroplastic changes. At the cellular level, neuroplastic changes are caused by altered neuronal excitability, synaptic efficacy, and subsequently increased or decreased firing patterns in neural circuits and networks [22]. Such changes are often caused by changed activity or signaling in the brain that, in turn, affect the gene expression of immune cells [23]. Also, neuro-physiological processes, such as the activation of the HPA axis and autonomic dysregulation, have also been found to be associated with this form of plasticity [24,25,26].

A non-invasive method for assessing brain activity is electro-encephalography (EEG). EEG offers a window into rapidly changing brain dynamics. EEG features such as changes in gamma band oscillations, amplitude, or latencies of so-called event-related potentials have previously been associated with acute and/or persistent pain [27]. By combining EEG recordings with a simple experimental manipulation of pain (the “tonic” cold pressor test—CP test [28]), previous studies have identified a number of EEG signatures of pain perception [29,30,31]. Thus, since experimental pain can be evoked by the CP test, and this pain has been shown to mimic clinical pain [19], it is possible to combine the CP test and EEG to assess the central processing of pain [28].

The central processing of pain often includes changes in the so-called “pain matrix” (e.g., the prefrontal cortex, amygdala, insula), which also affect the HPA axis and the ANS. The HPA axis and ANS play important roles in mediating bi-directional neural–immune interactions [32,33]. Moreover, it is known that maladaptive changes in HPA axis activity may lead to downstream effects such as increased circulating cortisol, which affects blood cell gene expression (mRNA synthesis) [34] and, thus, the function of circulating immune cells [35]. Moreover, both cortisol and the inflammatory molecules released from circulating immune cells (e.g., monocytes) cross, or at least affect the permeability of substances that could cross, the blood–brain barrier [35]. Therefore, maladaptive changes in the HPA axis and/or the ANS may affect the immune system, which, in turn, affects the CNS and creates a *vicious circle*, aiding in the development of persistent pain.

With persistent pain or prolonged psychological stress, i.e., prolonged HPA axis activation, suppression of the immune system has been observed [32,36,37]. This has been linked to pro-inflammatory effects in blood markers and functional changes in the CNS [34,38]. If this cycle is not broken, it has the potential to enhance or maintain this pain by upholding the HPA response and the increased levels of circulating cortisol levels, further impacting the immune system. One of the effects of cortisol that has been shown is changes in the gene expression profiles of peripheral blood mononuclear cells (PBMCs) in healthy individuals [39]. A link has also been found between persistent pain and changes in peripheral gene (mRNA) expression [40,41] and genes expressed in human brain tissues [42]. Hence, it is likely that physiological changes due to persistent LBP can be detected both centrally, e.g., brain activity, and peripherally, e.g., gene expression in PBMCs. 

Still, the predictive value of mapping the dynamic patterns of brain activity and blood cell mRNA expression for the “chronification” of pain remains to be investigated. Therefore, it is important to document any correlations between brain activity and peripheral RNA profiles. In other words, the reciprocal interaction between the CNS and the immune system may contribute to predict at-risk patients with a potential persistent LBP trajectory. In addition, the discovery of such predictive biomarkers may help to identify the core pathophysiological mechanisms important for the development of more informative examinations, novel therapeutics, and personalized medicine, i.e., a more cost-effective use of limited clinical resources.

### Main Objectives

The purpose of the present line of research is to better understand how the development of persistent pain may be associated with (1) changes in brain activity and (2) how this affects the gene expression profiles in blood. To achieve this, the current study aims to explore brain activity (measured with EEG) and gene expression profiles pre and post an acute stressful and painful intervention, i.e., the CP test, in three different pain populations (no pain, mild short-lasting, and moderate long-lasting pain). The specific objectives are:

1. To identify the key EEG features that can robustly distinguish the different pain states, rest vs evoked experimental pain (by the cold pressor test), and between individuals with different clinical pain statuses.
2. To examine potential gene expression changes before and after CP test within individuals with different clinical pain statuses.
3. To test for correlations between the EEG activity and blood mRNA profiles.
4. To investigate if an individual mRNA sequencing (seq) fingerprinting analysis can be used to predict at-risk patients with a possible persistent LBP trajectory.

## 2. Materials and Methods

This study is designed as an observational prospective cohort study. The first visit will be the only in-person visit, with follow-up questionnaires distributed to the participants via email at 2 weeks, 3 months, and 6 months. The participants will be recruited from the Auckland region, New Zealand. Ethical approval was obtained from the Health and Disabilities Ethics Committee (HDEC, reference: 2023 EXP 19096). No serious adverse events are expected for this study. However, some mild discomfort and soreness in the area of blood sampling might occur.

### 2.1. Participants

#### 2.1.1. Inclusion Criteria and Procedure

**Figure 1:**
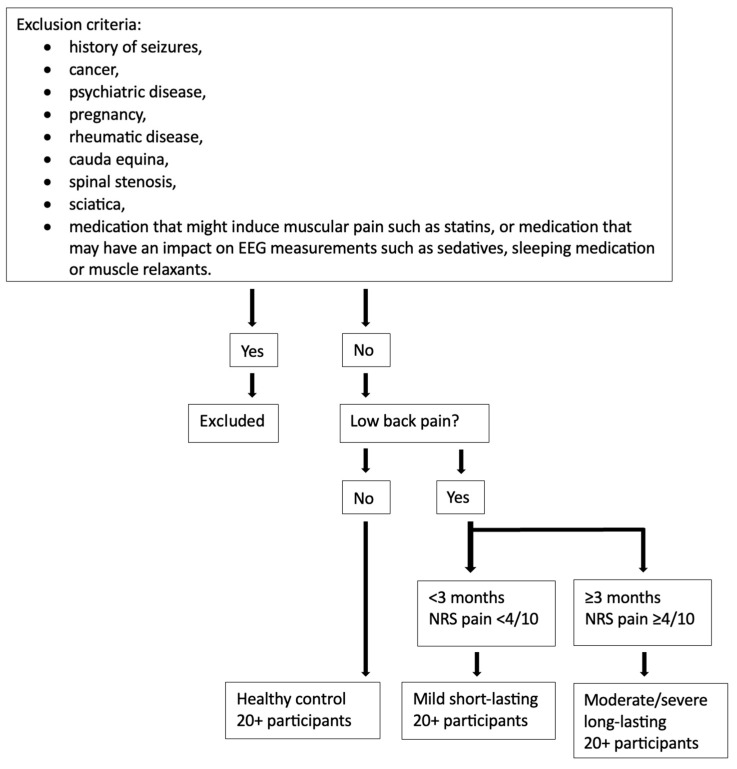
Proposed flow of participant exclusion criteria and group assignment. A total of at least 20 participants. EEG—electro-encephalography and NRS—numeric rating scale.

**Figure 2:**
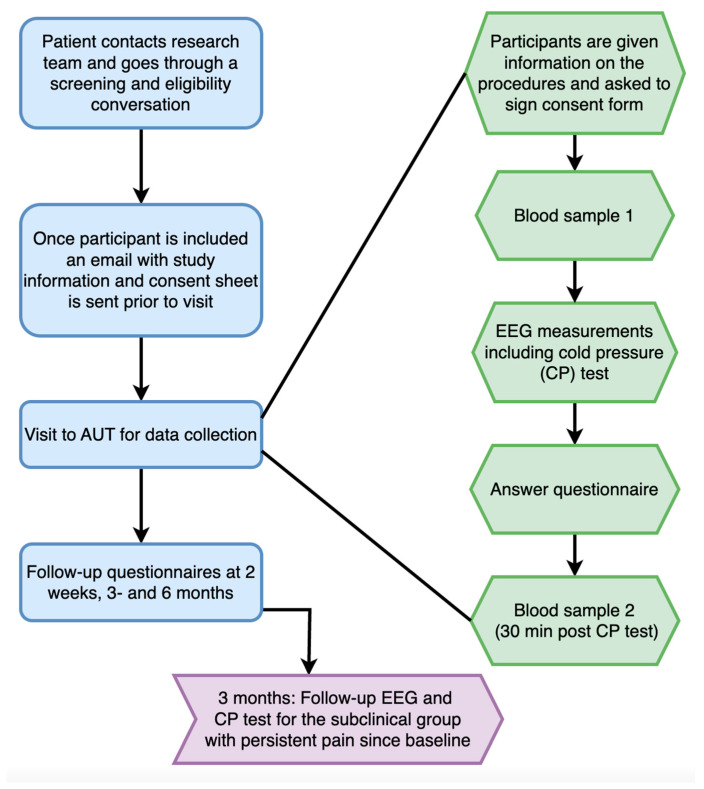
Proposed flow of study and data collection. AUT—Auckland University of Technology; EEG—electro-encephalography; CP—cold pressor. The EEG measurements consists of 3 min of eyes open and 3 min of eyes closed before and after CP test.

Participants of any gender between 18 and 50 years of age are invited to participate in the study. Advertisement and recruitment for the study will occur on social media (e.g., Facebook and Instagram), at outpatient clinics at hospitals in the Auckland region, and through flyers that will be posted at the surrounding educational centers, libraries, and other public spaces. Information on how to contact the research team for those interested in participation is provided on the flyers. Once the participant contacts the research team, one of the researchers will be in touch by phone or email. During this conversation, more information about the study will be provided, such as eligibility screening prior to inclusion and a more detailed description of the data collection procedure. This is also an opportunity for eligible participants to ask any questions and/or queries prior to accepting the invitation to participate in the study. The participants will also receive an email containing the consent form with the same information to read after the email/phone call. If participants have not contacted the research team within three days of receiving the consent form an email or a text message, they will be sent a reminder. On the day of the data collection, one of the researchers will again go through the screening sheet to ensure the correct group assignment and collect the signed consent forms before the participants are given the study questionnaire and blood sampling and EEG measurements begin. Participants will be assigned to either the healthy control group (no pain at time of inclusion), the mild short-lasting pain group (numeric rating scale (NRS) < 4/10 recurrent or persistent spinal ache, pain, or stiffness, lasting < 3 months), or the moderate/severe long-lasting pain group (NRS ≥ 4/10 recurrent or persistent non-specific LBP lasting ≥ 3 months). Figure 1 shows the proposed flow of the exclusion criteria and group assignment. The aim for the total number of participants in each group is set to at least 20 in each of the three groups. Figure 2 shows the proposed flow for the inclusion criteria and data collection.

Further, during the conversation on eligibility and inclusion, prior to the data collection, all participants will be informed about the purpose, benefits, and potential risks of participating. They will also receive information on their right to withdraw their participation at any time, without having to give a reason and without any consequence to them. All collected data (i.e., blood samples, EEG measurements, and questionnaires) will be de-identified prior to any pre-processing and/or analyses. 

#### 2.1.2. Exclusion Criteria

Participants who have a specific cause of LBP will be excluded. Also, if any potential participants have LBP due to pregnancy, rheumatic disease, cauda equina, spinal stenoses, and/or sciatica, they will not be able to participate in this study. Furthermore, if any potential participants have a history of seizures, cancer, psychiatric diseases, and/or are on any medications that may induce muscular pain such as statins or that have an impact on EEG measurements such as sedatives, sleeping medication, or muscle relaxants, they will also be excluded. 

### 2.2. Questionnaires

The baseline questionnaire will be provided digitally using a laptop set-up for the participants during the in-person visit. The participants will then receive links to follow-up questionnaires by email and will be asked to complete these questionnaires at 2 weeks, 3 months, and 6 months after the in-person visit. More information on the time frame for data collection and measurements is presented in Table 1.

#### 2.2.1. Demographic

Sociodemographic characteristic variables, measured only at baseline, include gender, age, marital status, ethnicity, education, employment, and dominant hand.

#### 2.2.2. Pain

The initial question regarding pain is a yes/no question about whether the participant currently is experiencing LBP. Thereafter, if the participants select yes, the following variables will be asked; current pain intensity measured by NRS [43], where 0 represents “no pain” and 10 represents “worst pain imaginable”, pain intensity average over the last week measured by NRS, duration of LBP, LBP trajectories (past 12 months and the next 12 months) [44], onset of LBP, perceived cause for LBP, and whether they are receiving treatment for their LBP (by general practitioner, chiropractor, physical therapist, or other). If the participants select no, they will be directed past the above-mentioned questions and to the question on whether they are experiencing pain elsewhere.

#### 2.2.3. Symptom Satisfaction and Expectation

Symptom satisfaction will be measured using the patient acceptable symptom state [45], a single item “How satisfied would you be if your current symptoms were to persist the rest of your life?”. This is measured by a 5-point Likert scale categorized into: “very satisfied”, “somewhat satisfied”, “neither satisfied nor dissatisfied”, “somewhat dissatisfied”, and “very dissatisfied”. Expectation will be measured with a single item “What is your expectation of recovery from back pain within 3 months?”, with a 5-point Likert scale categorized into: “complete recovery”, “somewhat better”, “no change”, “somewhat worse”, and “worse I’ve ever been”.

#### 2.2.4. Secondary Variables

To measure the psychological and functional aspects of experiencing pain, fear avoidance, pain catastrophizing, and functional status, the Fear-Avoidance Questionnaire (physical activity) [46,47], Pain Catastrophizing Scale [48], and Roland–Morris Disability Questionnaire [49] are included, respectively. To measure sleep, the 6-item Bergen Insomnia Scale will be used [50]. Questions regarding pain medication use for LBP, comprising type, dosage, and frequency, in addition to the Prescription Drug Use Questionnaire [51], are included. Further, to assess mental health, the Hopkins Symptom Check List—10 [52] and the Beck’s Depression Inventory will be used [53]. 

#### 2.2.5. EEG Measurement

The EEG will be recorded at a sampling rate of 1024 Hz from 62 channels using an REFA amplifier (TMSi, Twente, The Netherlands) according to the 10–20 electrode system. The reference electrodes will be placed on the right and left mastoids (M1 and M2, respectively), while the ground electrode will be placed at AFz. These channels are shown in Table 2. The impedance of the electrodes will be kept below 10 kΩ. The subjects will be asked to focus on a fixation cross with a plain background displayed in the center of a whiteboard while minimizing their eye blinks, eye movements, and facial movements. Additionally, online filter settings will be adjusted to a range of DC-100 Hz.

**Figure 3:**
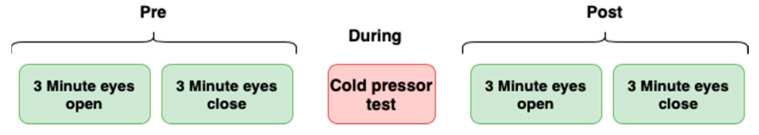
EEG data collection protocol.

The preparation for the EEG will take around 20 min, following which, the resting-state EEG will be recorded at three different time points: before, during, and after the CP test. During the pre and post recordings, the EEG will be measured while the participant has their eyes open for 3 min, followed by 3 min recorded with their eyes closed, resulting in a total of 12 min of EEG data recording. When the EEG is recorded during the CP test, the participant will immerse their hand in ice water for 80 s (CP test). The EEG data collection protocol is shown in Figure 3. Participants from the mild short-lasting pain group who report persistent pain between baseline and 3 months will be invited for a second EEG measurement and CP test shortly after the 3-month time point for follow-up.

#### 2.2.6. Cold Pressor (CP) Test

The CP test will be performed using a circulating water bath (Grant, Fischer Scientific, Slangerup, Denmark). The water will be cooled to 2 °C and the subjects will immerse their left or right hand in the water up to the wrist for 80 s.

#### 2.2.7. mRNA Sequencing

A total of two blood samples will be collected for each participant, pre and 30 min post CP test. The blood sample collection will be performed by a registered nurse or other qualified personnel using the BD vacutainer Push Button Blood Collection Set and BD vacutainer Green Lithium Heparin tubes (10 mL). After the blood sample collection, peripheral blood mononuclear cells (PBMC) will be isolated from the sample (using SepMate (STEMCELL Technologies, Vancouver, BC, Canada) see File S1 for full protocol). To preserve the samples, a freezing medium will be added (using Cryostor CS10 (STEMCELL Technologies, Vancouver, BC, Canada), see File S2 for full protocol). Thereafter, the samples will be shipped to the commercial company Novogene Co., Ltd., Cambridge, UK, for mRNA isolation and sequencing. 

### 2.3. Statistical Analyses

#### 2.3.1. Descriptive Analyses at Baseline

Categorical variables will be presented as percentages, whereas continuous variables will be presented as means and standard deviations. Descriptive analyses will be presented for the entire data set. A comparison will be made between the groups on demographic characteristics in addition to primary outcomes to assess for selection bias. Regression models (linear, logistic, and/or ordinal, depending on outcome) at baseline will be conducted to assess for group differences at baseline. 

To assess the clinical course of the primary outcome variable, multilevel models will be used. For the statistical evaluation of differences across groups and changes over time, linear mixed or generalized linear mixed regression models will be set up for continuous outcomes, and ordinal mixed regression models will be used for ordinal outcomes. All these models will have a longitudinal analysis of covariance structure, in which post-test outcome means and odds ratios will be presented. Moreover, within-participant correlations arising from repeat measurements will be controlled for by estimating participant-wise random intercepts and/or participant-wise slopes across time. The choice of the final model structure will be decided by minimizing the information loss quantified by Akaike’s Information Criterion (AIC). The effect sizes for the baseline-adjusted between-group differences and within-group changes over time estimated from the models will be reported along with their 95% confidence intervals.

Moreover, a prediction model will be created to assess the gene expression profiles as predictors for the main outcomes. This will be conducted using three-step backward stepwise regression analyses; (1) gene expression profiles and other variables deemed to be of importance will be used to fit a regression model with which the Akaike information criterion (AIC) will be calculated, (2) the variable with the least significant *p*-value will be removed. Step 2 will be repeated until the removal of any prognostic factors no longer affects or increases the AIC or all *p*-values are <0.157 [54]. R-squared values will be used to assess the overall model performance. Area under the curve (AUC) will be used to assess discriminating ability (values of 0.7 to 0.8 are considered acceptable, 0.8 to 0.9 excellent, and 0.9 to 1.0 outstanding) [55]. The Hosmer–Lemeshow test will be used to estimate the calibration and calibration slope (*p*-value > 0.05 is considered good calibration). Internal validation will also be conducted using 200 bootstrap samples. 

#### 2.3.2. Power Calculation

**Figure 4:**
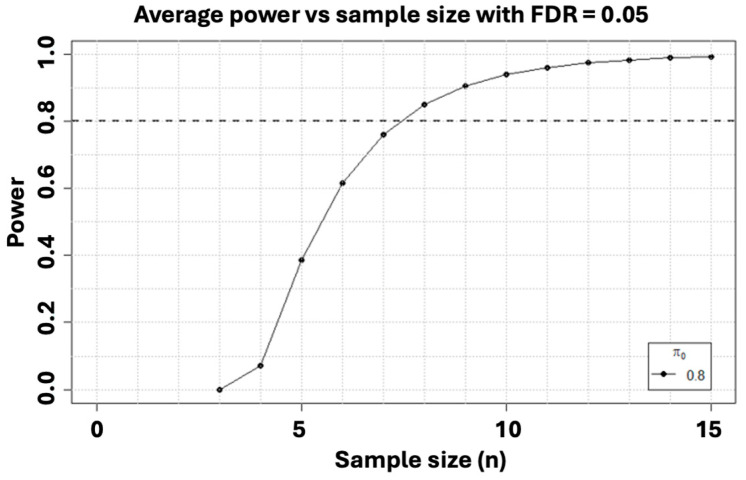
Preliminary Ahus data from our own group. Power calculation based on mRNA seq analyses (3 pain free + 3 participants with pain) showing a required 8 + 8 participants in each group to ensure sufficient statistical power of 0.95, *p* < 0.01.

To assess the sample size requirements for the genetic aspect of the study, a power calculation was conducted. Current mRNA sequencing analyses showed that 8 test subjects versus 8 controls should provide a significant difference regarding the gene expression (see Figure 4). This calculation was based on pilot data from a previous project including three pain free patients and three pain patients. Hence, aiming for at least 20 participants in each group is estimated to suffice.

#### 2.3.3. EEG Processing

The raw EEG data will be preprocessed offline using EEGLAB (version 14.1.1) [56] and ERPLAB (version 6.1.4) [57] running on MATLAB (2015b) (the MathWorks, Inc, Natick, MA, United States). For the EEG preprocessing, 62 electrodes will be used for the data processing, whereas the average of mastoids (M1 and M2) will be used as a reference. The PREP pipeline (version 0.55.1) [58] will be used to remove and interpolate bad channels, line noise, and re-referencing. The following PREP pipeline parameters will be used. Line frequencies of 50 Hz and their harmonics will be selected for noise removal while keeping the taper bandwidth and window size/step as their default settings. We will select the robust average referencing method for the PREP pipeline. The spline interpolation method will be used to interpolate bad channels highlighted by the PREP pipeline. This pipeline will try to interpolate bad channels, and very noisy channels will be left as “still noisy channels”. These “still noisy channels” will be removed from the data and adaptive mixture independent component analyses (AMICA) will be run. After the completion of the AMICA, the removed channels will be interpolated back into the data using spline interpolation. 

**Figure 5:**
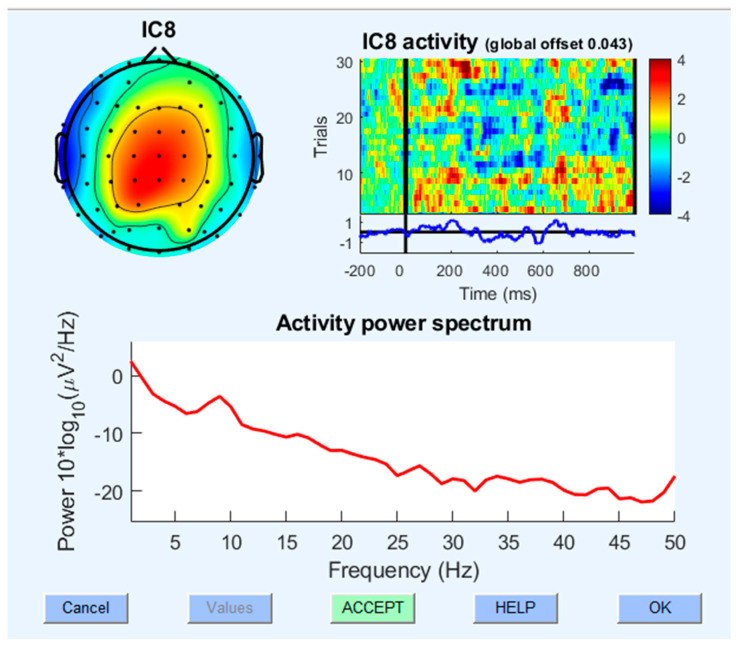
Different features of independent components. To ensure the results are actual brain components, features such as 1/f frequency response, clear dipole formation, and activity resembling random brain activity will be used.

After running the PREP pipeline, the data-cleaning steps highlighted in a previous study [59] will be followed. The IClabel [60] will be used to mark the AMICA components into brain, eye, muscle, channel, and other noise. These markings will then be visually inspected based on the components’ features such as frequency response, activity window, and dipole formation (see Figure 5). 

**Figure 6:**
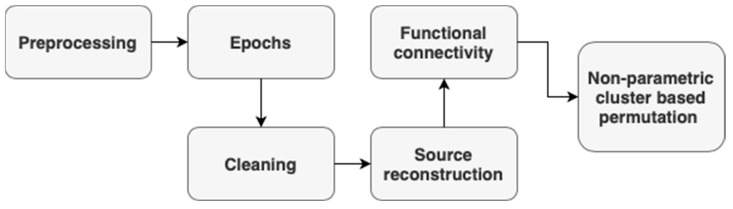
EEG processing pipeline.

After using IClabel and manual checks, the data will then be cleaned and loaded into Brainstorm [61] for source estimation and EEGLAB [56] for frequency-based analysis. The complete EEG processing pipeline is shown in Figure 6.

#### 2.3.4. EEG Source Localization

**Figure 7:**
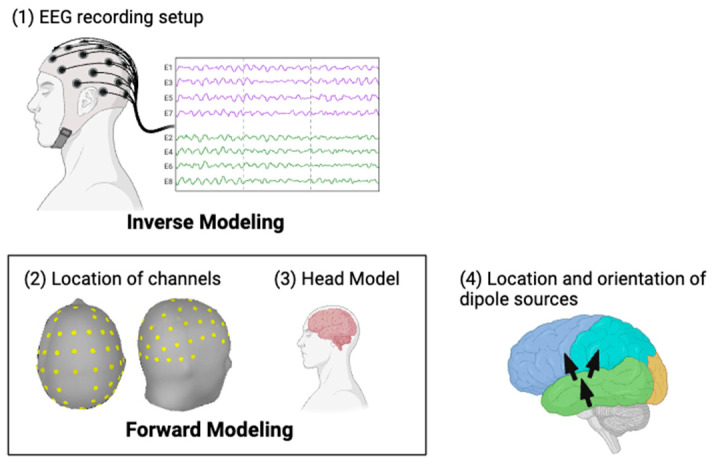
Source reconstruction. Made with BioRender.

To estimate the location and activity of underlying neural sources based on measurements obtained from multiple sensors or electrodes [62], EEG source reconstruction will be performed using Brainstorm [61] in MATLAB R2022a. The overall process of source reconstruction is shown in Figure 7. 

There are two main problems in EEG source reconstruction: forward modeling and inverse modeling. Both are dependent on each other for accurate source reconstruction. Forward modeling involves the human head, including its scalp, skull, cortex, and electromagnetic properties, as shown in Figure 7. The inverse modeling problem uses information about cortical activity from forward modeling. 

##### Forward Modelling

This section outlines the forward modeling process for EEG source reconstruction. The goal in forward modeling is to determine the location and orientation of EEG sensors relative to the cortical source, which requires defining the location and orientation of the current dipole fields [61,63]. This will be accomplished by placing source dipoles on a voxel grid space approximating the cortical space, ensuring that the orientation is perpendicular to the cortex. The symmetric boundary element method (Open MEEG BEM) will be used to model the dipoles for all subjects [61]. A default generic head model from Brainstorm will be employed, which features 15,000 vertices and a three-layer compartment (scalp, skull, and brain). Tissue conductivities will be set based on a previous study [63]: scalp = 1, skull = 0.0125, and brain = 1. The forward model will be calculated after defining the 64 electrode locations, including M1 and M2, on the scalp using the 10–12 electrode placement system and the 64-channel location file of the TMSI 64-channel amplifier.

##### Inverse Modelling

For the inverse modeling, the standardized low-resolution brain electro-magnetic tomography (sLORETA) method will be used to adjust the current density maps of the source dipoles, representing them as normalized current densities perpendicular to the cortex [64]. To efficiently assess functional connectivity, high-resolution sources will be grouped based on the Desikan–Killiany atlas, which defines 68 regions of interest (ROIs) on the cortex surface. Averaging the time series within each ROI, a [ROIs × time] matrix will be formed. The sign of the dipoles will be flipped in the opposite direction before averaging to prevent activity cancellation. This approach will enable an accurate estimation of brain activity and an understanding of how different brain regions are connected.

Once source reconstruction is completed, a functional connectivity analysis will be calculated based on the Phase Lag Index (PLI). The data will be divided into narrow-band signals. A fourth-order Butterworth filter will acquire the three frequency bands, alpha, beta, and gamma. The frequency bands of the EEG sources of the brain will be defined from the reported ranges: alpha (7.5–12.5 Hz), beta (12.5–30 Hz), and gamma (30–40 Hz) [65]. To create 68 × 68 connectivity matrices, the Desikan–Killiany atlas of 68 regions will be used. Out of the 68 regions of the Desikan–Killiany atlas, brain areas will be selected based on their previous association with the perception of pain, including but not limited to the sensory cortex, anterior cingulate cortex, and prefrontal cortex [66]. The focus will be on the three brain networks associated with pain: (1) Default Mode Network (DMN), (2) Central Executive Network (CEN), and (3) Salience Network (SEN). The associated brain regions of these networks in the Desikan–Killiany atlas are shown in Table 3. 

These connectivity matrices (68 × 68) will be loaded into GraphVar 2.0 [67] along with the brain source information from Table 2 for a cluster-based permutation test to identify significant connectivity patterns within specified brain regions. These significant brain regions will be plotted on the cortex using BrainNet viewer 1.7 [68].

##### Cluster-Based Permutation Using GraphVar

In the present study, the GraphVar toolbox will be used to analyze Phase Lag Index (PLI) connectivity matrices [67]. The connections between each pair of nodes within the matrix will be examined. To handle the challenge of multiple comparisons, GraphVar organises significant links into Graph-Components, which can be considered as sub-networks. These components will be measured, such as how clusters are identified in fMRI [69]. GraphVar compares these to randomly generated data within the software to determine if a graph component’s size is non-random. GraphVar then computes a *p*-value for each non-random component. This will allow GraphVar to pinpoint significant connectivity patterns. In the statistical section of GraphVar, the within-subject design will be chosen, where data from subjects will be collected across multiple sessions (before, during, and after CP test) coupled with a between-group analysis. GraphVar then calculates the mean PLI differences between two sessions simultaneously (e.g., pre–post) and between groups. Importantly, these calculations will only consider significant non-random graph components. The results will highlight the brain connections where the main PLI is significantly different between sessions, hence identifying changes in connectivity patterns.

In summary, GraphVar will be provided with a 68 × 68 PLI matrix and 31 regions of interest. In its output, GraphVar will provide a 31 × 31 probability matrix and a 31 × 31 effect size matrix, which will then be loaded into BrainNet Viewer [68] for visualization. 

#### 2.3.5. EEG Frequency and Time–Frequency Analysis

The clean data from all sensors (electrodes) will be used for the frequency analysis. The average power of each classical frequency band, including delta (1–4 Hz), theta (4.1–8 Hz), alpha (8.1–12 Hz), beta (12.1–32 Hz), and gamma (32.1–80 Hz) will be determined. These measurements will be used to analyze differences in the basic EEG frequency-based parameters among different conditions (rest, CP test) and groups (control, short-lasting, and long-lasting pain). Additionally, correlations between these measurements and key demographic and clinical variables will be assessed. This approach will help to identify potential alterations in theta, alpha, and beta power, as previous studies have shown this in these frequency bands during experimentally induced pain and a trend towards a decreased alpha and beta power in persistent musculoskeletal pain conditions. A time–frequency analysis will also be performed to investigate the dynamic changes in brain oscillations during rest and experimentally induced pain conditions. 

To investigate the dynamic changes in brain oscillations, a time–frequency analysis on the pre-processed EEG data will be performed. The data will first be divided into 20 s epochs (broader analysis). The power spectrum obtained from each epoch’s Fast Fourier Transform (FFT) will then be multiplied by a set of complex Morlet [70] wavelets through a process called convolution. This allows for a simultaneous examination of the power of the EEG signals at different frequencies and time points. Morlet wavelets, characterized by sinusoidal waves modulated by a Gaussian envelope, can capture dynamic changes in brain oscillations across different frequency bands (e.g., delta, theta, alpha, beta, and gamma) by observing how the power of these frequency bands varies over time within each epoch. This method effectively reveals how brain activity evolves temporally and across different frequencies.

#### 2.3.6. mRNA Sequencing Analyses

Quality control and analyses of the gene expression data will be conducted as follows. The quality of the raw sequencing files will first be assessed with fastqc (v0.11.9) and multiqc (v1.11), and adapters together with the low-quality bases will be removed using trimmomatic (v0.39). The trimmed reads will be mapped to the hg38 reference human genome with the STAR aligner (v2.7.9a) [71], and postprocessing of the mapped reads will be performed with samtools (v1.13) [72]. The gencode annotation corresponding to the reference genome will be used to summarize the read counts across the relevant genomic features (e.g., genes, exons, and promoters) with featureCounts (included in subread package v2.0.1) [73]. The obtained read counts will be used for the further downstream phase of the expression data analysis. In the next phase of the data analysis, DESeq2 (v1.32.0) [74] will be used to identify differences in the expression profiles between the patient groups. A gene set enrichment analysis will be performed with clusterProfiler (v4.0.0) [75], and Weighted Gene Co-expression Network Analysis (WGCNA) (v1.70-3) [76] will be used to identify groups of co-expressing genes, as well as to explore relatedness between clinical variables and the groups of co-expressing genes.

## 3. Expected Results

Recruitment will start during the second quarter of 2024 and is expected to run for 6 to 9 months, with the final data collection completed within the first quarter of 2025. The results are expected to be ready by the third quarter of 2025. 

Upon the completion of this study, the expected results will (1) identify the key EEG features that can distinguish different pain states, before and after evoked experimental pain (CP test), (2) uncover potential gene expression changes before and after CP test within and between individuals with different clinical pain statuses, (3) test if there is a correlation between the EEG activity and mRNA profiles of the individuals, and (4) investigate if the uncovered mRNA profiles can be used to predict a higher risk of a persistent LBP trajectory. We hope that the combination of these methods can provide more knowledge on the transition from acute to persistent pain, which, in turn, could guide future research focus. 

## 4. Discussion

In this paper, we presented a protocol describing the design and methods for an unexplored combination of modalities using EEG and RNA seq of blood cells for investigating the transition from mild short-lasting (acute) to moderate/severe long-lasting (persistent) LBP. We hypothesize that this combination may reveal correlations between EEG, RNA seq, and pain that can help to predict the development of persistent pain. 

The initial aspect of the project will focus on establishing the groundwork, i.e., mapping any EEG features that can be seen in different pain states, and uncovering mRNA expression profiles. These findings will then be assessed in relation to each other and the outcomes. A comparison will also be made between the RNA profiles before and after the CP test with the intent to investigate any resulting changes induced by the test. Further, these results will then be used to determine if the mRNA expression profiles can be used as predictors for an increased risk of persistent LBP. For those in the mild short-lasting pain group who are invited back for a second round of EEG, a comparison of brain activity between baseline and the 3-month follow-up will be conducted.

Despite EEG being a non-invasive and relatively simple method to use, it has some challenges, such as, e.g., EEG source reconstruction. For accurate source reconstruction, forward and inverse modeling are dependent on each other [63]. Forward modeling involves the human head, including its scalp, skull, cortex, and electromagnetic properties [77]. Inverse modeling uses the information about cortical activity from forward modeling to identify the most likely locations and strength [63]. 

Regardless, persistent LBP is famously a complex condition. Hence, understanding the different influencing physiological factors leading to persistent LBP, as well as those maintaining its vicious cycle, is paramount. This study will, therefore, be an important first step in assessing whether the chosen modalities are compatible in assessing such processes. By exploring both neuronal activity and gene expression as potential predictors, the intention is to explore alternative ways of uncovering predictors and increasing the understanding of the physiological processes involved in persistent LBP. 

### 4.1. Limitations

As with all longitudinal studies, compliance may be a challenge. Due to the chosen distribution of questionnaires, via email, there is a risk of participants forgetting to answer. If an answer is not received within a couple of days, participants will be contacted by telephone with a gentle reminder. However, by distributing the questionnaires via email and not asking participants to meet up in person, this may lower the effort needed to respond, as the questionnaire can be answered on the participants’ own laptop or mobile device at their leisure.

Another challenge could be the time between the CP test and the second blood sample collection. Currently, there is a lack of peer-reviewed evidence providing guidelines regarding optimal time between stimuli and measurable changes in blood mRNA expression. However, previous studies have collected blood samples at different time points from immediately after their test protocol up to 20, 30, and 40 min and even 8 h [33,78,79]. The post-CP-test sample for the present study will be taken at 30 min based on these previous studies and also for pragmatic reasons. We do, however, acknowledge that this may be a weakness that could directly influence the chance of detecting a measurable change in gene expression peripherally.

Further, by including a potential second in-person assessment, there might be some of those invited who will decline the invitation. Additionally, as it is expected that only a small number of the mild short-lasting group will develop persistent pain, the number of participants invited back might be small. Still, the first part of the study has an acceptable power. Also, the study will be important for the design of further studies. 

### 4.2. Data Management, Storage, and Security

The collected data from the questionnaires and EEG measurements will be stored on institutional network drives with firewalls and security measures in place according to national and European Union data protection regulations. Any hard copy records will be locked in a secure location for storage. Any access to these records is limited to selected study personnel. The collected blood samples will be de-identified prior to PBMC processing and stored in a −80 °C freezer at AUT Roche Laboratory. The storage room is secured by locked doors and is only accessible to approved staff at AUT Roche Laboratory. The samples will be stored in this room safely until shipment to Novogene, Co., Ltd., Cambridge, UK, for further analyses. Biological material that is not used for the mRNA sequencing will be destroyed according to the current regulations at AUT Roche Laboratory. Only personnel directly involved in the sample collection will have access to the collected material. All collected data will be de-identified and stored separately from the raw data. For the purpose of analyses, only anonymized and de-identified data will be used. 

## Figures and Tables

**Table 1:** Content of the questionnaires and collection times.

Demographics	Baseline	2 Weeks	3 Months	6 Months
Gender	X			
Age	X			
Marital status	X			
Ethnicity	X			
Education	X			
Employment	X			
Dominant hand	X			
Currently experiencing low back pain ^a^	X	X	X	X
Current severity of low back pain (11-point numeric rating scale)	X ^a^	X ^a^	X ^a^	X ^a^
Severity of low back pain average last week (11-point numeric rating scale)	X ^a^	X ^a^	X ^a^	X ^a^
Duration of current low back pain	X ^a^	X ^a^	X ^a^	X ^a^
Low back pain trajectory last 12 months	X ^a^	X ^a^	X ^a^	X ^a^
Symptom satisfaction (PASS 5-point Likert scale)	X ^a^	X ^a^	X ^a^	X ^a^
Onset of low back pain	X ^a^	X ^a^	X ^a^	X ^a^
Perceived cause of low back pain	X ^a^	X ^a^	X ^a^	X ^a^
Receiving treatment for low back pain	X ^a^	X ^a^	X ^a^	X ^a^
Fear Avoidance Belief Questionnaire—physical activity	X ^a^	X ^a^	X ^a^	X ^a^
Pain Catastrophizing Scale	X ^a^	X ^a^	X ^a^	X ^a^
Roland–Morris Disability Questionnaire	X ^a^	X ^a^	X ^a^	X ^a^
Expectation of recovery within 3 months	X ^a^	X ^a^	X ^a^	X ^a^
Low back pain trajectories next 12 months	X ^a^	X ^a^	X ^a^	X ^a^
Current pain elsewhere	X	X	X	X
Bergen Insomnia Scale	X	X	X	X
Use of pain medication for low back pain ^b^	X	X	X	X
Type of medication	X ^b^	X ^b^	X ^b^	X ^b^
Name of medication	X ^b^	X ^b^	X ^b^	X ^b^
Administration method for medication	X ^b^	X ^b^	X ^b^	X ^b^
Medication use frequency	X ^b^	X ^b^	X ^b^	X ^b^
Medication dosage	X ^b^	X ^b^	X ^b^	X ^b^
Medication use duration	X ^b^	X ^b^	X ^b^	X ^b^
Prescription Drug Use Questionnaire	X ^b^	X ^b^	X ^b^	X ^b^
Hopkins Symptom Check List—10	X	X	X	X
Beck’s Depression Inventory	X	X	X	X

^a^—further questions will only appear if the answer is yes on the question “Currently experiencing low back pain”. ^b^—further questions will only appear if answer is yes on the question “use of pain medication for low back pain”. All variables are self-reported. For the questionnaire, the primary outcomes measures are: (1) LBP intensity measured by 11-point numeric rating scale (NRS), (2) symptom satisfaction measured by patient acceptable symptom state (PASS), and (3) expectation of recovery within 3 months. These variables will be especially important for uncovering self-reported persistent LBP trajectory which, in turn, will be analyzed in combination with the EEG measurement and the mRNA expression profile.

**Table 2:** Sixty-four channel names according to 10–20 system.

FP1	CP6	FC4
FPz	P7	C5
FP2	P3	C1
F7	PZ	C2
F3	P4	C6
FZ	P8	CP3
F4	POZ	CPZ
F8	O1	CP4
FC5	OZ	P5
FC1	O2	P1
FC2	AF7	P2
FC6	AF3	P6
T7	AF4	PO5
C3	AF8	PO3
CZ	F5	PO4
C4	F1	PO6
T8	F2	FT7
CP5	F6	FT8
CP1	FC3	TP7
CP2	FCZ	TP8
PO7	PO8	M1
M2		

**Table 3:** Desikan–Killiany brain regions forming DMN, CEN, and SEN.

bankssts (left)	parsorbitalis (left)
caudalanteriorcingulate (left)	parsorbitalis (right)
caudalanteriorcingulate (right)	posteriorcingulate (left)
frontalpole (left)	posteriorcingulate (right)
frontalpole (right)	precuneus (left)
inferiorparietal (left)	precuneus (right)
inferiorparietal (right)	rostralanteriorcingulate (left)
insula (left)	rostralanteriorcingulate (right)
insula (right)	rostralmiddlefrontal (left)
isthmuscingulate (left)	rostralmiddlefrontal (right)
isthmuscingulate (right)	superiorparietal (left)
lateralorbitofrontal (left)	superiorparietal (right)
lateralorbitofrontal (right)	supramarginal (left)
medialorbitofrontal (left)	supramarginal (right)
medialorbitofrontal (right)	
parahippocampal (left)	
parahippocampal (right)	

## Data Availability

Once collected and processed, data may be available on request.

## Supplementary Materials

The following supporting information can be downloaded at: https://www.mdpi.com/article/10.3390/brainsci14070641/s1, File S1: Peripheral blood mononuclear cell (PBMC) isolation protocol. File S2: Cryopreservation protocol.
