# Supplementary material for: Investigating Whether a Combination of Electro-Encephalography and Gene Expression Profiling Can Predict the Risk of Chronic Pain: A Protocol for an Observational Prospective Cohort Study
Source: Brain Sci. 2024 Jun 26;14(7):641. doi: 10.3390/brainsci14070641 (PMC11274615; doi:10.3390/brainsci14070641)
Supplement: Supplementary file 1 [file brainsci-14-00641-s001.zip › brainsci-3022867-supplementary.pdf]

File S1

This document is available at www.stemcell.com/PIS

SepMate™

SepMate™-50

h #85450	100 Tubes
h #85460	500 Tubes

SepMate™-15

h #85415	100 Tubes
h #85420	500 Tubes

STEMCELL TECHNOLOGIES
Scientists Helping Scientists™ | WWW.STEMCELL.COM
TOLL FREE PHONE 1 800 667 0322 • PHONE +1 604 877 0713
INFO@STEMCELL.COM • TECHSUPPORT@STEMCELL.COM
FOR GLOBAL CONTACT DETAILS VISIT OUR WEBSITE

ENGLISH

Intended Use

SepMate™ is used to isolate mononuclear cells (MNCs, comprising lymphocytes and monocytes) from human whole blood or bone marrow by density centrifugation. For in vitro diagnostic use.

Product Description

MNCs are commonly isolated by density centrifugation. With this method, defibrinated or anticoagulant-treated blood is carefully layered on a density gradient medium and centrifuged for a short period of time. Differential migration during centrifugation results in the formation of layers containing different cell types. The bottom layer contains erythrocytes which have been aggregated by the density gradient medium and therefore sediment completely through the density gradient medium. The layer immediately above the erythrocyte layer contains mostly granulocytes, which at the osmotic pressure of the density gradient medium solution attain a density great enough to migrate through the density gradient medium layer. Because of their lower density, the MNCs are found at theinterface between the plasma and the density gradient medium with other slowly sedimenting particles (platelets). The MNCs are carefully recovered from the interface and washed.

The specialized insert in SepMate™ minimizes mixing of the sample and the density gradient medium, thereby avoiding the need for careful layering and careful cell removal from the interface. Density gradient medium is pipetted through a central hole in the insert, partially filling the tube. Whole blood is then rapidly pipetted down the side of the tube to rest upon the density gradient medium. After centrifugation for 10 minutes with the brake on, the enriched cell layer is simply poured off into a new tube, while the density gradient medium, erythrocytes, and granulocytes are retained below the insert. The MNCs are washed and are then ready for use.

Microbial State

SepMate™ tubes were irradiated by an electron beam process that conforms to the applicable requirements of ISO 11137-1. Do not use if the integrity of the packaging is compromised. Do not re-use.

Storage and Stability

Store at ambient temperature. Product stable at ambient temperature until expiry date on label.

Warnings and Precautions

1. For in vitro diagnostic use by professional laboratory users only. Not for use with therapeutic applications.
2. SepMate™ is single use. Do not re-use.
3. Do not use SepMate™ tubes after the expiry date indicated on the label.
4. Do not use SepMate™ tubes if the product or the packaging is damaged or compromised.
5. SepMate™ is not intended for a specific diagnostic application. Validating SepMate™ for a specific diagnostic downstream application, includingusing SepMate™ in combination with other reagents (e.g. RosetteSep™ cocktails), is the responsibility of the end user.
6. This product should be handled by trained personnel observing good laboratory practices. Once the SepMate™ tube contains sample, it should be treated as potentially

biohazardous. Dispose of tubes and biological waste in accordance with appropriate local, state, or national biohazard safety regulations.

7. SepMate™ can be used with human whole peripheral blood and bone marrow samples. It is not intended for use with leukapheresis samples, buffy coat samples, or samples older than 48 hours.
8. Centrifuge tubes at recommended settings.
9. Following centrifugation, cells may aggregate on the SepMate™ tube wall above the MNC layer. This aggregation is normal and is influenced by sample quality and age, and type of anticoagulant used. This aggregation is not related to the use of SepMate™. The cells can be dislodged by usinga pipette tip to scrape the side of the tube.

Materials Required but Not Provided

Laboratory Reagents

- Density gradient medium such as Lymphoprep™ (Catalog #07801) or any similar medium with a density of 1.077 g/mL designed for the separation of mononuclear cells.
- Dulbecco's Phosphate Buffered Saline with 2% Fetal Bovine Serum (PBS + 2% FBS; Catalog #07905). Laboratory

Equipment

- Low-speed centrifuge with a swinging bucket rotor
- Serological pipettes (e.g. Catalog #38004)

Sample Preparation

Collect whole blood using an appropriate anticoagulant (such as acid-citrate-dextrose [ACD] or heparin). Whole blood specimens may be stored at roomtemperature (15 - 25°C) for no more than 48 hours before use with SepMate™ following the Directions for Use.

Collect bone marrow using an appropriate anticoagulant (such as ACD or heparin). Bone marrow specimens may be stored at room temperature for no more than 48 hours before use with SepMate™ following the Directions for Use.

Directions for Use

Ensure that sample, recommended medium (PBS + 2% FBS), density gradient medium (see Materials Required but Not Provided), and centrifuge are all at roomtemperature (15 - 25°C).

1. Add density gradient medium to the SepMate™ tube by carefully pipetting it through the central hole of the SepMate™ insert. Refer to Table 1 for required volumes. The top of the density gradient medium will be above the insert.

   NOTE: Small bubbles may be present in the density gradient medium after pipetting. These bubbles will not affect performance.
2. Dilute sample with an equal volume of PBS + 2% FBS. Mix gently.

   For example, dilute 5 mL of sample with 5 mL of PBS + 2% FBS.
3. Keeping the SepMate™ tube vertical, add the diluted sample by pipetting it down the side of the tube. The sample will mix with the density gradientmedium above the insert.

   NOTE: The sample can be poured down the side of the tube. Take care not to pour the diluted sample directly through the central hole.
4. Centrifuge at 1200 x *g* (see Notes) for 10 minutes at room temperature, with the brake on. NOTE: For

   samples older than 24 hours, a centrifugation time of 20 minutes is recommended.

   NOTE: Different makes and models of centrifuges may provide different rates of deceleration when braking. If a layer of MNCs is not visible following centrifugation or the recovery of MNCs is low, reduce the rate of deceleration (i.e. braking) to medium or low.
5. Pour off the top layer, which contains the enriched MNCs, into a new tube. Do not hold the SepMate™ tube in the inverted position for longer than2 seconds.

   NOTE: Some red blood cells (RBCs) may be present on the surface of the SepMate™ insert after centrifugation. These RBCs will not affectperformance.

   NOTE: To reduce platelet contamination in the enriched MNCs, pipette off some of the supernatant above the MNC layer before pouring.
6. Wash enriched MNCs with PBS + 2% FBS. Repeat wash.

   NOTE: Centrifuging at 300 x *g* for 8 minutes at room temperature, with the brake on, is recommended.

   NOTE: To remove platelets from the enriched MNCs, perform one of the washes at 120 x *g* for 10 minutes at room temperature, with the brake off.

   NOTE: If the density gradient medium above the SepMate™ insert appears red after centrifugation (i.e. some RBCs have not pelleted), the SepMate™ tube can be spun at 1200 x *g* for another 10 minutes with the brake on. This step may be necessary when processing samples that are older than 24 hours.

Table S1. Sample and Density Gradient Medium Volumes

SEPMATE™ TUBE	INITIAL SAMPLE (mL)	DENSITY GRADIENT MEDIUM (mL)
15	0.5 - 4	4.5
15	> 4 - 5	3.5
50	4 - 17	15

Protocol Diagram

Numbers in brackets refer to steps under Directions for Use.

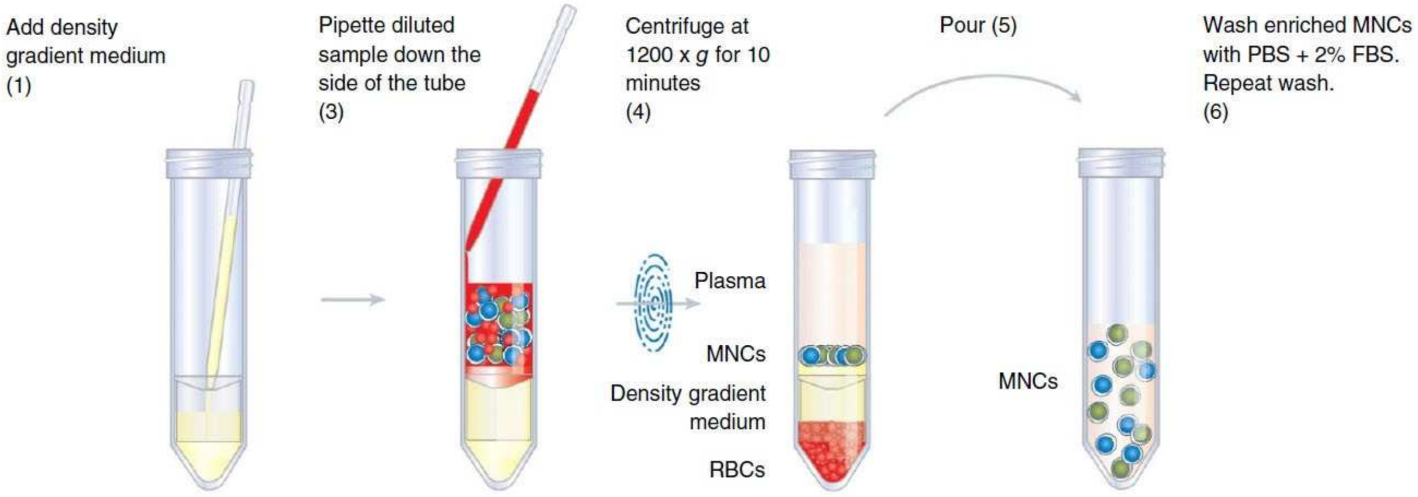

Notes

SepMate™-15

SepMate™-15 is designed to process 0.5 - 5 mL of initial sample.

A minimum packed RBC volume of 0.25 mL is required. For samples with low hematocrits, the minimum sample volume may therefore be greater than 0.5 mL.There is a maximum packed RBC volume of 3 mL. For samples with very high hematocrits, the maximum sample volume may therefore be less than 5 mL. SepMate™-50

SepMate™-50 is designed to process 4 - 17 mL of initial sample.

A minimum packed RBC volume of 2 mL is required. For samples with low hematocrits, the minimum sample volume may therefore be greater than 4 mL. There is a maximum packed RBC volume of 12 mL. For samples with very high hematocrits, the maximum sample volume may therefore be less than 17 mL.

Conversion of *g* to RPM

To convert *g* to RPM, use the following formula:

$$\text{RPM} = \sqrt{\frac{\text{RCF}}{(1.118 \times 10^{-5}) \times (\text{Radius})}}$$

Where: RPM = centrifuge speed in revolutions per minuteRCF =
relative centrifugal force (*g*)
Radius = radius of centrifuge rotor in centimeters (cm)

Technical Assistance

For technical support, contact techsupport@stemcell.com or call toll-free either +1.604.877.0713 (Canada), +1.800.667.0322 (North America), 00800 7836 2355 (Europe), or +1.800.060.350 (Australia). For more information, visit www.stemcell.com. If you require a printed copy or a translated version of this document in acertain language, contact us at techsupport@stemcell.com.

Any serious incident that has occurred in relation to the device shall be reported to the manufacturer and, if the user is located within the European Union, thecompetent authority of the Member State in which the user is established.

Deletions, additions, or changes are indicated by the change bar in the margin.

h<br>Catalog or reference number	g<br>Batch code	H<br>Use by: YYYY-MM-DD
Y<br>Caution, consult accompanying documents	V<br>In Vitro Diagnostic Medical Device	Do not use if packaging is damaged
C<br>CE Mark	M<br>Manufacturer's identification (name & address)	EC REP<br>Authorized EC representative in the European Community
Consult Instructions for Use	Do not re-use	Σ<br>Contains sufficient forn tests

SepMate™ Catalog #85460/85420 are shipping configurations for 500 tubes that use products from SepMate™ Catalog #85450/85415 (100 tubes). The Declaration of Conformity and technical documentation for Catalog #85450/85415 are applicable to these shipping configurations (Catalog #85460/85420).

Copyright © 2023 by STEMCELL Technologies Inc. All rights reserved including graphics and images. STEMCELL Technologies & Design, STEMCELL Shield Design, Scientists Helping Scientists, SepMate, and RosetteSep are trademarks of STEMCELL Technologies Canada Inc. All other trademarks are the property of their respective holders. While STEMCELL has made all reasonable efforts to ensure that the informationprovided by STEMCELL and its suppliers is correct, it makes no warranties or representations as to the accuracy or completeness of such information.

File S2

CryoStor® CS10

Animal component-free, defined cryopreservation medium with 10% DMSO

Catalog #	
07959	5 x 10 mL
07952	16 x 10 mL
07931	5 x 16 mL
07930	100 mL
100-1061	100 mL
07955	100 mL Bag
07940	1000 mL Bag

STEMCELL™ TECHNOLOGIES
Scientists Helping Scientists™ | WWW.STEMCELL.COM
TOLL FREE PHONE 1 800 667 0322 • PHONE +1 604 877 0713
INFO@STEMCELL.COM • TECHSUPPORT@STEMCELL.COM
FOR GLOBAL CONTACT DETAILS VISIT OUR WEBSITE

Product Description

CryoStor® CS10 is a uniquely formulated, serum-free, animal component-free, and defined cryopreservation medium containing 10% dimethylsulfoxide (DMSO). Designed to preserve cells in low-temperature environments (-70 to -196°C), CryoStor® CS10 provides a safe, protective environment for cells and tissues during the freezing, storage, and thawing processes. CryoStor® CS10 is recommended for the cryopreservation of hepatocytes, tissue samples, blood-derived cells, CHO cells, myeloma cell lines, hybridomas, human mesenchymal stem cells, human embryonic stem (ES) and induced pluripotent stem (iPS) cells, and other extremely sensitive cell types.

- Ready-to-use
- Serum-free, protein-free
- Animal component-free
- cGMP manufactured with USP grade/highest-quality components
- FDA master file
- Sterility, endotoxin, and cell-based quality control testing

Product Information

CATALOG #	SIZE	STORAGE	SHELF LIFE	CONTAINS
07959	5 x 10 mL	Store at 2 - 8°C.	Stable until expiry date (EXP) on label. Protect from prolonged exposure to light.	10% DMSO
07952	16 x 10 mL	Store at 2 - 8°C.	Stable until expiry date (EXP) on label. Protect from prolonged exposure to light.	10% DMSO
07931	5 x 16 mL	Store at 2 - 8°C.	Stable until expiry date (EXP) on label. Protect from prolonged exposure to light.	10% DMSO
07930	100 mL	Store at 2 - 8°C.	Stable until expiry date (EXP) on label. Protect from prolonged exposure to light.	10% DMSO
100-1061*	100 mL	Store at 2 - 8°C.	Stable as noted in the Stability Memo.** Protect fromprolonged exposure to light.	10% DMSO
07955	100 mL Bag	Store at 2 - 8°C.	Stable for 2 years from date of manufacture (MFG) onlabel. Protect from prolonged exposure to light.	10% DMSO
07940	1000 mL Bag	Store at 2 - 8°C.	Stable for 2 years from date of manufacture (MFG) onlabel. Protect from prolonged exposure to light.	10% DMSO

* New bottle type; for more information, contact us at techsupport@stemcell.com.

** Refer to the Stability Memo on the product page.

Refer to the Safety Data Sheet (SDS) for hazard information.

Product may be shipped at room temperature (15 - 25°C) and should be refrigerated upon receipt.

Handling/Directions for Use

CRYOPRESERVING CELLS

For cryopreserving human embryonic stem (ES) cells and induced pluripotent stem (iPS) cells, refer to the Technical Manuals for mTeSR™1 ormTeSR™ Plus for further information, available at www.stemcell.com, or contact us to request a copy.

1. Wipe down the outside of the CryoStor® CS10 container with 70% ethanol or isopropanol before opening.
2. Obtain a cell suspension using a cell-specific protocol and centrifuge cells to obtain a cell pellet.
3. Carefully remove the supernatant with a pipette, leaving a small amount of medium to ensure the cell pellet is not disturbed.Resuspend the cell pellet by gently flicking the tube.
4. Add cold (2 - 8°C) CryoStor® CS10, mix thoroughly, and transfer the suspension to a cryovial.
5. Incubate cells at 2 - 8°C for 10 minutes.
6. Cryopreserve cells using a standard slow rate-controlled cooling protocol (approximately -1°C/minute) or an isopropanol freezingcontainer, and store at liquid nitrogen temperature (-135°C).

   NOTE: Long-term storage at -80°C is not recommended.

THAWING CELLS

1. Warm medium of choice in a 37°C water bath.
2. Wipe the outside of the vial of cells with 70% ethanol or isopropanol.
3. In a biosafety cabinet, twist the cap a quarter-turn to relieve internal pressure and then retighten.
4. Quickly thaw cells in a 37°C water bath by gently shaking the vial. Do not submerge the vial. Remove the vial when only a small frozencell pellet remains. Do not vortex cells.
5. Wipe the outside of the vial with 70% ethanol or isopropanol.
6. Dilute cells 1 in 10 with warmed medium.
7. Centrifuge the cell suspension at 300 x *g* for 10 minutes at room temperature (15 - 25°C).
8. Carefully remove the supernatant with a pipette, leaving a small amount of medium to ensure the cell pellet is not disturbed.Resuspend the cell pellet by gently flicking the tube.
9. Gently add medium to the tube.

CRYOSTOR PRODUCTS MEET USP <71> STERILITY AND USP <85> ENDOTOXIN TESTING STANDARDS, AND ARE MANUFACTURED UNDER CGMP.

Copyright © 2022 by STEMCELL Technologies Inc. All rights reserved including graphics and images. STEMCELL Technologies & Design, STEMCELL Shield Design, and Scientists Helping Scientists are trademarks of STEMCELL Technologies Canada Inc. CryoStor is a registered trademark of BioLife Solutions. mTeSR is a trademark of WARF. All other trademarks are the property of their respective holders. WhileSTEMCELL has made all reasonable efforts to ensure that the information provided by STEMCELL and its suppliers is correct, it makes no warranties or representations as to the accuracy or completeness of such information.
